# Opposite Roles of Bacterial Cellulose Nanofibers and Foaming Agent in Polyhydroxyalkanoate-Based Materials

**DOI:** 10.3390/polym14245358

**Published:** 2022-12-07

**Authors:** Mădălina Gabriela Oprică, Cătălina Diana Uşurelu, Adriana Nicoleta Frone, Augusta Raluca Gabor, Cristian-Andi Nicolae, Valentin Vasile, Denis Mihaela Panaitescu

**Affiliations:** 1 National Institute for Research and Development in Chemistry and Petrochemistry, 202 Splaiul Independentei, 060021 Bucharest, Romania; 2 Cantacuzino National Medical-Military Institute for Research and Development, 103 Splaiul Independentei, 050096 Bucharest, Romania

**Keywords:** polyhydroxybutyrate, foam, nanocellulose, thermal properties, DMA

## Abstract

In this work, an economically feasible procedure was employed to produce poly (3-hydroxybutyrate-*co*-3-hydroxyvalerate) (PHBV)-based foams. Thermally expandable microspheres (TESs) were used as a blowing agent, while bacterial cellulose (BC) nanofibers served both as a reinforcing agent and as a means of improving biocompatibility. PHBV was plasticized with acetyltributylcitrate to reduce the processing temperature and ensure the maximum efficiency of the TES agent. The morphological investigation results for plasticized PHBV foams showed well-organized porous structures characterized by a porosity of 65% and the presence of both large pores (>100 µm) and finer ones, with a higher proportion of pores larger than 100 µm being observed in the PHBV nanocomposite containing TESs and BC. The foamed structure allowed an increase in the water absorption capacity of up to 650% as compared to the unfoamed samples. TESs and BC had opposite effects on the thermal stability of the plasticized PHBV, with TESs decreasing the degradation temperature by about 17 °C and BC raising it by 3–4 °C. A similar effect was observed for the melting temperature. Regarding the mechanical properties, the TESs had a flexibilizing effect on plasticized PHBV, while BC nanofibers showed a stiffening effect. An in vitro cytotoxicity test showed that all PHBV compounds exhibited high cell viability. The addition of TESs and BC nanofibers to PHBV biocomposites enabled balanced properties, along with lower costs, making PHBV a more attractive biomaterial for engineering, packaging, or medical device applications.

## 1. Introduction

The pressing need to find alternatives to petroleum-derived products has made room for the development of “green” materials via innovative procedures to relieve environmental challenges. A promising branch in the polymeric industry is represented by biopolymers, produced from renewable sources, with poly(lactic acid) and polyhydroxyalkanoates (PHAs) among the most popular [1,2,3]. PHAs are synthesized by Gram-positive bacteria, Gram-negative bacteria, archaea, and microalgae [2,4]. Some of the most attractive members of the PHA family are poly(3-hydroxybutyrate) (PHB), poly (3-hydroxybutyrate-*co*-3-hydroxyvalerate) (PHBV), and poly(3-hydroxybutyrate-*co*-3-hydroxyhexanoate) (PHBH) [3,5,6]. The incorporation of hydroxyvalerate (HV) units into PHB during biosynthesis has an important effect on the mechanical properties, crystallization rate, and crystallinity of the resulting copolymer, which is characterized by better ductility, lower crystallinity, easier melt processing, and increased biodegradability [2,3,5]. Its good compatibility with human cells makes PHBV suitable for medical applications such as tissue engineering, wound dressings, surgical sutures, and drug delivery systems [7]. Moreover, PHBV-based materials have found applications in active packaging [8], electromagnetic interference shielding [9], and green electronic components [10].

The growing interest in PHBV has made it possible to find solutions to its drawbacks. An important shortcoming of PHB and PHBV is their brittleness, which needs to be corrected in order to expand their production and application. A currently applied solution is pairing the brittle biopolymer with a plasticizer, which allows the material to be processed more easily, improving its ductility and elongation at break [11]. In accordance with ecological rules, bio-based plasticizers such as glycerol, glycerol triacetate, and citric acid derivatives have been developed and intensively studied as modifiers for brittle biopolymers [11,12]. The good compatibility of fatty acid esters with PHBV and better properties in terms of improved impact strength and resistance to aging were reported for PHBV plasticized with fatty acid esters [11]. The best results were obtained when lauric acid ethylene glycol monoester, palmitic acid methyl ester, and oleic acid methyl ester were used as plasticizers in PHBV. Although HV units act as an internal plasticizer for PHB in the PHBV copolymer, external plasticization using citrate esters in particular proved to be much more effective [11,13,14]. However, the addition of plasticizers, especially in high amounts, which is necessary for improved flexibility, damages other important properties, such as permeability and strength [11,12,13]. The concomitant addition of a reinforcing agent of natural origin, such as cellulose, and a bio-based plasticizer has been proposed as a solution in many works, mainly for their application in food packaging [15,16].

Cellulose is a renewable and biodegradable polysaccharide, accessible all around the world, and has found its way into polymeric nanocomposites via nanocellulose (NC) [2]. The main problem in the case of wood-derived nanocellulose is the deforestation needed for its production [2]. This issue and the need to avoid competition with food sources led to the investigation of other sources of nanocellulose, such as agro-industrial waste, whose exploitation for the isolation of nanocellulose is about to become a widely applied technology [17]. Another solution is bacterial cellulose (BC), a biopolymer synthesized by aerobic bacteria, particularly by bacteria belonging to *Komagataeibacter* sp. [18,19]. This type of cellulose is produced in the form of exopolysaccharides (extracellular polymers) and has the same molecular formula as plant-derived cellulose [2]. Regarding its properties, BC stands out thanks to its high crystallinity, high tensile strength, better structural properties, and porosity, together with higher purity as compared to plant-based cellulose [2,20,21]. The high purity of BC comes from the lack of its association with lignin, pectin, and hemicelluloses, which are present in plant cell walls; this gives it a fair advantage when used in biomedical applications due to the lack of toxicity and side effects [20]. BC with improved mechanical properties, good porosity, and better stability in physiological conditions was obtained by impregnating BC and surface-modified BC sponges with PHBV [22]. BC nanofibers were also mixed with PHBV powder using a high-energy ball-milling technique and compression molding to improve the barrier properties of PHBV [23]. Multilayer composites showing reduced water vapor permeability were obtained from BC membranes impregnated with glycerol or polyethylene glycol and coated with a PHBV solution in formic acid [2].

Melt-compounding techniques, which are environmentally friendly and easy to scale up, have rarely been used for the preparation of PHB/BC or PHBV/BC nanocomposites [24,25,26]. This is mostly due to the difficulty of ensuring a good dispersion of BC nanofibers in the polyalkanoate matrix during melt compounding. To enhance the dispersion of the nanofiller and other specific properties, a bioelastomer (poly (3-hydroxyhexanoate-*co*-3-hydroxyoctanoate) was added to the PHB/BC composition [24] or a salt-leaching technique was employed, resulting in porous scaffolds [25]. Recently, the use of a blowing agent in PHBV/BC nanocomposites led to porous structures showing improved ductility and lower consumption of materials, along with lower costs [26]. 

In the last few years, different blowing procedures have been used to reduce PHBV’s brittleness and production costs [7,25,27,28]. Particulate leaching is a popular technique often used to obtain high-porosity biopolymer foams for biomedical purposes, but it is rarely employed to obtain PHBV foams due to the chlorinated derivatives used as solvents and the extensive washing required by this technique [7,25]. The use of supercritical (sCO_2_) or subcritical carbon dioxide as a physical blowing agent has become a preferred option in recent years due to its nontoxicity and nonflammability [28]. PHBV/polycaprolactone blend foams with open and closed cells were obtained using subcritical CO_2_ as a blowing agent [28]. However, the porosity and open-cell proportion decreased with the increase in the proportion of PHBV in the blends. The main problems that arise when blowing the PHBV melt using CO_2_ are (i) the high crystallinity of PHBV, which leads to the need for high temperatures for complete melting, (ii) its low melt strength, which favors cells’ collapse and coalescence, and (iii) low CO_2_ dissolution in PHBV [29]. These problems can be at least partially eliminated by using thermally expandable microspheres (TESs), which acted as good blowing agents for PHBV [26]. TESs consist of a low-boiling-point hydrocarbon (e.g., isopentane) contained inside a thermoplastic shell layer that expands over a delimited temperature interval. Thus, cell collapse is prevented due to the shell layer. In addition, TESs have an advantage over chemical blowing agents due to their ability to better create reproducible spherical cavities [30].

However, foaming PHBV with TESs has the disadvantage of working at the upper temperature limit of the blowing agent. Therefore, in this work, TESs were used as a blowing agent in PHBV plasticized with acetyltributylcitrate (ATBC) to reduce the PHBV processing temperature and ensure the maximum efficiency of the thermally expandable microspheres. BC nanofibers were also added as a reinforcement to improve the properties of foamed PHBV. The effects of BC nanofibers and the TES blowing agent in the plasticized PHBV-based formulations were determined in this work by means of thermogravimetric analysis (TGA), differential scanning calorimetry (DSC), and dynamic mechanical analysis (DMA). The porous structure of the nanocomposite foams was characterized by scanning electron microscopy (SEM) and, indirectly, by water absorption ability. Unfoamed and foamed PHBV products were evaluated for their in vitro cytotoxicity by using the MTT assay. The foamed biomaterials obtained by the method proposed here can be applied in the automotive and aeronautical industries, as well as in the biomedical and packaging fields due to their light weight, strength, and biodegradability. 

## 2. Materials and Methods

### 2.1. Materials

Poly-(3-hydroxybutyrate-*co*-3-hydroxyvalerate) (PHBV) with 12% poly-(3-hydroxyvalerate) content was supplied by Goodfellow Cambridge Ltd. (Huntingdon, UK) in granular form with a density of 1.250 g cm^−3^ and served as a matrix. The TES blowing agent was purchased from Nouryon (Sundsvall, Sweden) under the trade name of Expancel 920 DU 120. The plasticizer, acetyl tributyl citrate, was supplied by Sigma Aldrich (St. Louis, MO, USA).

Fresh bacterial cellulose pellicles containing about 1% cellulose and 99% water [31] were produced under static conditions by a *Komagataeibacter xylinus* strain and mechanically disintegrated as previously reported [26]. Briefly, bacterial cellulose nanofibers, denoted as BC, were obtained by defibrillating the pellicles with a high-speed blender for 30 min and, after being diluted with distilled water (1:1, *w*/*w*), with an LM20 Microfluidizer (Microfluidics, Westwood, MA, USA) for twelve passes. The resulting BC suspension in water containing about 0.5 wt% BC was frozen at −20 °C for 48 h and freeze-dried using a FreeZone 2.5 L equipment (Labconco, Kansas City, MO, USA).

### 2.2. Preparation of PHBV Nanocomposite Foams

TES microspheres (Expancel 920 DU 120) consist of a methacrylate copolymer shell carrying about 20% isopentane blowing agent and can be expanded between 130 and 200 °C. Mixing PHBV with 10 wt% ATBC plasticizer, 2 wt% BC nanofibers, and 3 wt% TES microspheres took place in the W50 EHT mixing chamber of a Brabender Plasti-Corder LabStation (16 KW, 400 Nm/350 min^−1^, Brabender GmbH & Co., Duisburg, Germany) at a rotor speed of 40 min^−1^. To keep the TES microspheres unexpanded in the mixing stage, a specific temperature–time profile was followed: PHBV granules were melted and mixed with the plasticizer at 150 °C for about 3 min, then the temperature was lowered to 120–125 °C (therefore, below the expansion temperature), and the microspheres carrying the blowing agent were added. Mixing continued at this temperature for a total time of 10 min. For the samples containing BC, the nanofibers were added to the melted polymer together with the plasticizer before the temperature drop and the addition of the blowing agent. Further, plasticized PHBV (denoted as PHBV), plasticized PHBV reinforced with BC (denoted as PHBV/BC), plasticized PHBV containing expandable microspheres (denoted as PHBV-E), and plasticized PHBV reinforced with BC and containing expandable microspheres (denoted as PHBV/BC-E) were processed by compression molding into rectangular bars with dimensions of 70 mm × 10 mm × 4 mm. A Collin press (Maitenbeth, Germany) was used for this purpose in the following conditions: temperature of 175 °C, preheating for 150 s, and compression for 75 s. This temperature was selected to ensure the maximum efficiency of TESs. The set pressure for the compression step was 5 MPa for PHBV and PHBV/BC and 0.5 MPa for the samples containing the expandable microspheres. Rapid cooling for 2 min in a cooling cassette completed the compression-molding process and prevented the shrinkage of the foamed specimens.

### 2.3. Characterization

#### 2.3.1. Scanning Electron Microscopy (SEM)

The morphological characteristics of the PHBV composite foams were analyzed by SEM using a Hitachi TM4000 plus microscope (Hitachi, Tokyo, Japan) at an accelerating voltage of 15 kV. Prior to the SEM analysis, the unfoamed and foamed rectangular bars were fractured in liquid nitrogen and sputter-coated with a thin layer (5 nm) of gold using a Q150R Plus (Quorum Technologies, SXE, Lewes, UK). The pore diameter and cell density were analyzed using at least three SEM micrographs with the aid of ImageJ software Version 1.8.0 (NIH, Bethesda, Rockville, MD, USA). About 200 pores were analyzed for each of the foamed samples to calculate the average pore size (*d_c_*) according to the following equation [32]:

(1) $$d_{c}=\;\frac{\sum_{i=1}^{n}d_{i}}{n}$$

where *d_i_* and *n* are the diameter and the number of cells in the micrograph. 

#### 2.3.2. Thermal Characterization

A thermogravimetric analysis was carried out using a TGA Q5000 from TA Instruments (New Castle, DE, USA). The measurements were taken under a nitrogen flow of 40 mL min^−1^ with a heating rate of 10 °C min^−1^ in the temperature range from 25 °C to 700 °C. 

A DSC Q2000 (TA Instruments, New Castle, DE, USA) was used to investigate the melting and crystallization behaviors of the PHBV compositions. The three steps of the DSC analysis (heating, cooling, and heating) were carried out under a helium flow as follows: raising the temperature from –55 °C to 205 °C in the first step, temperature equilibration for a few minutes, then dropping the temperature to –55 °C in the cooling step, and heating up to 205 °C in the final step. The melting temperatures in the first (T_m_(I)) and second cycles (T_m_(II)) and the crystallization temperature (T_c_), along with the corresponding enthalpies, were determined from the obtained curves. The degree of crystallinity was calculated from the second melting cycle, after the thermal history was erased, as the ratio between the melting enthalpies corresponding to both peaks and the product between the melting enthalpy of 100% crystalline PHBV (109 J g^−1^ [33]) and the mass fraction of PHBV in the compounds, multiplied by 100.

#### 2.3.3. Dynamic Mechanical Analysis

The mechanical properties of unfoamed and foamed samples were determined using a dynamic mechanical analyzer, DMA Q800, from TA Instruments (New Castle, DE, USA). The measurements were taken on parallel bar samples with a length × width × thickness of 60 mm × 10 mm × 4 mm at a frequency of 1 Hz using a dual cantilever clamp. The bar samples were heated at 3 °C min^−1^ from −50 °C to 155 °C. 

#### 2.3.4. Water Absorption

The water uptake of the samples was measured until equilibrium was reached by immersing the dry samples (*W*_0_) in distilled water at room temperature for 96 h. Three rectangular bars of 10 mm × 10 mm × 4 mm (length × width × thickness) from each sample were used for these measurements. At selected intervals, the samples were removed from the water, wiped with filter paper to remove excess water, weighed, and reimmersed in water. This protocol was repeated until no further weight change was observed. The water uptake (*W*) was calculated with the following equation [34]:

(2) $$W\%=\frac{W_{i}-W_{0}}{W_{0}}\times 100$$

where *W*_i_ is the wet weight of the samples at time i.

#### 2.3.5. Evaluation of In Vitro Cytotoxicity

The cytotoxic effect of the samples on the L929 cell line was evaluated using the MTT assay. L929 murine fibroblast cells were cultured in DMEM (Dulbecco’s Modified Eagle Medium, Lonza, Verviers, Belgium) supplemented with 10% fetal bovine serum (FBS, Biochrom AG, Berlin, Germany), 100 U/mL penicillin, and 100 μg/mL streptomycin (Lonza, Verviers, Belgium). Samples (about 5 mg) representing small sections of the rectangular bars obtained by compression molding were incubated for 24 h at 37 °C in 1 mL of serum-free culture medium under sterile conditions. The extracts were filtered through a 0.22 μm microfilter (Sartorius Stedim Biotech, Goettingen, Germany). L929 cells were seeded in 96-well plates at a density of 10^4^ cells/well in a humidified atmosphere at 37 °C and 5 % CO_2_ and allowed to adhere overnight in complete culture medium (100 μL/well). Afterward, the culture medium was replaced with 100 μL/well of undiluted sample extracts and a dilution series of the extracts (50.00%, 25.00%, 12.50%, 6.25%, 3.13%, 1.57%, 0.78%, and 0.39%). The viability of L929 cells after 72 h exposure was quantitatively assessed using the MTT assay. Thus, the medium was collected from each well, and MTT solution (100 μL containing 90 μL of complete culture medium and 10 μL of MTT) was added to each well and then incubated for 3 h at 37 °C. Later, the cells were lysed, and the extinction was determined using a microplate reader spectrophotometer (TecanSunrise ^TM^, Groedig, Austria) at a 570 nm wavelength. Cells grown in complete culture medium served as the control. Cell viability was calculated as the ratio between the absorbance of the sample and the absorbance of the control, multiplied by 100. Each eluate and the control were tested in triplicate wells. 

#### 2.3.6. Detection of Endotoxin

The Limulus amebocyte lysate (LAL) chromogenic endpoint assay (HIT302, Hycult Biotech, Uden, The Netherlands) was used to determine the concentration of Gram-negative bacterial endotoxin in the PHBV compounds. Thus, samples of the medium corresponding to undiluted extracts were tested following the LAL assay protocol. This is based on the fact that in the presence of endotoxins, an enzyme extracted from amebocytes of the American horseshoe crab (*Limulus polyphemus*) causes yellow coloring due to the cleavage of p-nitroaniline. The reaction was stopped by the addition of acetic acid, and the absorbance at 405 nm was measured with a spectrophotometer. 

## 3. Results and Discussion

### 3.1. Morphological Analysis

**Figure 1:**
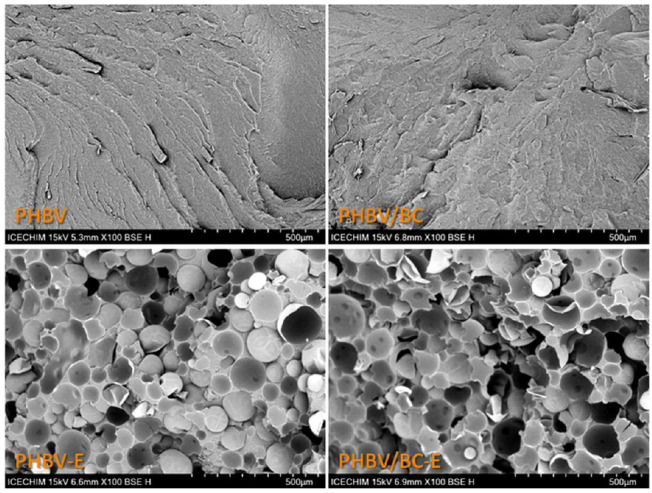
SEM images of PHBV, PHBV/BC, and corresponding foamed products.

**Figure 2:**
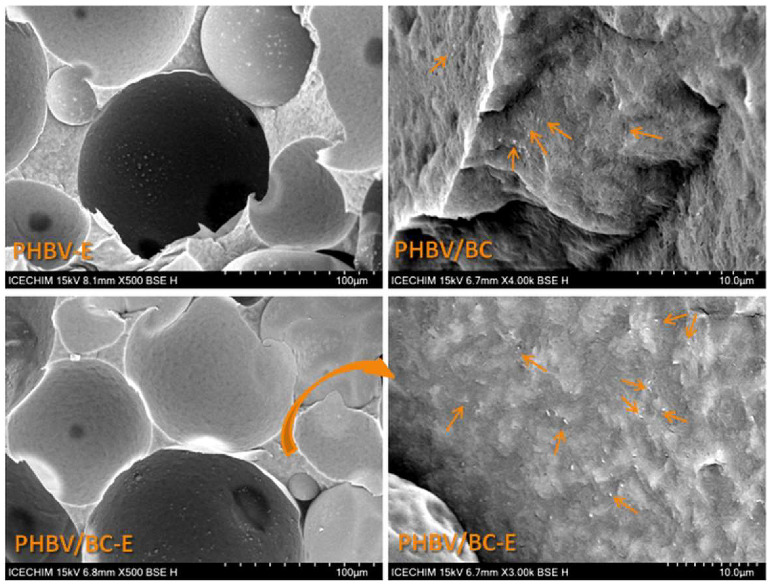
SEM images of PHBV-E, PHBV/BC, and PHBV/BC-E; the BC nanofibers are marked with arrows.

Figure 1 shows representative SEM images of the fractured surfaces of PHBV, PHBV/BC, and their foamed counterparts. The fractured surface of PHBV displays an ordered arrangement due to the high crystallinity of PHBV [26]. The addition of BC disturbs, to some extent, this orderly structure, as observed in the fractured section of PHBV/BC. The higher-magnification SEM image (Figure 2) of the fractured surface of the PHBV/BC nanocomposite shows a very good dispersion of BC nanofibers in the PHBV matrix. The nanofibers have thicknesses generally varying between 50 and 100 nm. 

The SEM images of the PHBV and PHBV/BC foams presented in Figure 1 show microcellular structures. The pores are relatively homogeneous in size and dispersion in the section of the matrix in both PHBV-E and PHBV/BC-E foams. This is a remarkable feature of the foams obtained with TESs, which is not generally found in the case of PHBV foams obtained by other methods, such as in those using supercritical carbon dioxide [9] or blowing agents [35]. These methods lead to irregular cellular structures showing coalescent and ruptured pores as a result of the poor melt strength of PHBV [9]. This PHBV deficiency is prevented in PHBV- and PHBV/BC-E by using thermally expandable microspheres as a blowing agent.

**Figure 3:**
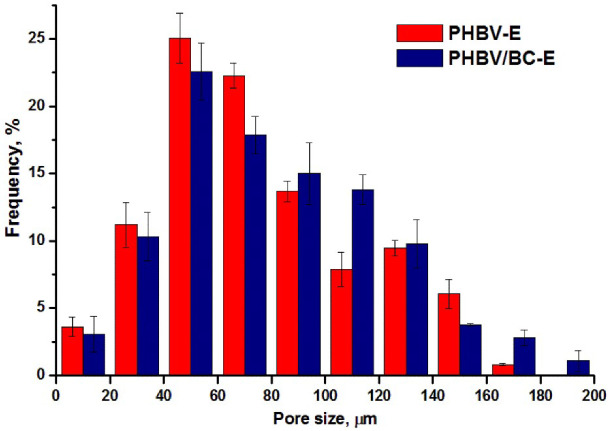
Pore size distribution of foamed samples; data are presented as mean ± standard error.

A quantitative analysis of the pore size and porosity of the foamed samples was carried out using ImageJ and about 200 pores from three SEM images of each sample. The results are presented in Table 1 and Figure 3. The average pore sizes in Table 1 are presented as means ± standard errors at a 99% confidence level. 

Although the addition of 2 wt% BC nanofibers could slightly increase the melt viscosity of PHBV, contributing to a lower porosity and pore size, the data in Table 1 show that the average pore size was greater for the nanocomposite as compared to PHBV-E, and the porosity was similar for the two foamed samples. Therefore, due to their nanodimensions and good dispersion, the BC nanofibers had no obvious influence on the porosity of plasticized PHBV, but they slightly influenced its porous structure, as observed in Figure 3. 

A higher proportion of larger pores was noticed in the nanocomposite as compared to PHBV-E. Thus, in the PHBV-E sample, 25.16 ± 2.96% of the pores were larger than 100 µm, while in PHBV/BC-E, more than 30% of the pores (31.22 ± 0.84%) had sizes >100 µm. Although the difference was not statistically significant (*p* > 0.05), it can be observed that the cellulose nanofibers did not hinder the foaming process. 

**Figure 4:**
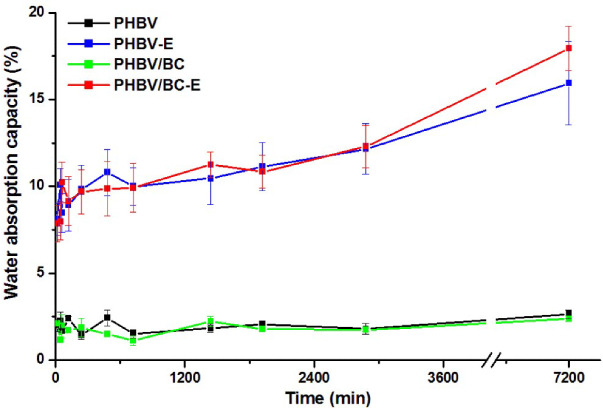
Water absorption of unfoamed and foamed PHBV samples.

Variable proportions of open cells and holes are observed in the section of both foamed samples in Figure 1 and Figure 2. To better emphasize the presence of open cells, the water absorption of the samples was studied, knowing that a higher content of open cells generally increases the absorption of water [37]. The water absorption variation with time for all samples for a short period of time of only five days is given in Figure 4. 

For the PHBV and PHBV/BC samples, the water uptake was low (around 2%) and showed insignificant variation with time. Indeed, PHBV is a hydrophobic polymer with a low water absorption capacity. However, PHBV-E and PHBV/BC-E showed 640 and 650 % increases in water uptake as compared to the unfoamed samples after five days of immersion in water. This demonstrates the presence of open cells in both foamed samples. 

### 3.2. Thermogravimetric Analysis

**Figure 5:**
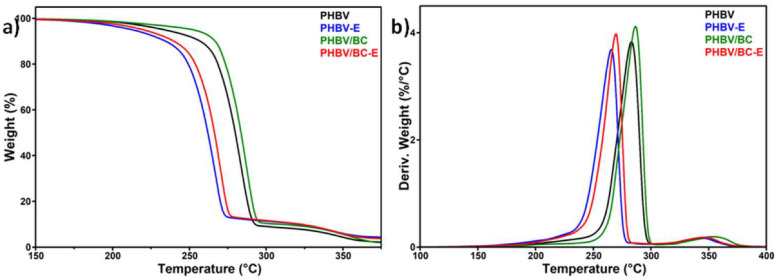
Thermogravimetric (**a**) and derivative (**b**) curves of PHBV-based materials.

The changes in the thermal stability of plasticized PHBV caused by the addition of nanocellulose and the blowing agent can be observed in Figure 5, which presents the TGA and DTG curves of the compounds. PHBV-based materials containing the blowing agent were the first to suffer thermal degradation: the onset degradation temperature (T_on_) was 247.7 °C for PHBV-E and 253.7 for PHBV/BC-E, while T_on_ was 19.1 °C and 17.6 °C higher, respectively, for the corresponding unfoamed materials (Table 2). 

Contrary to the blowing agent, BC nanofibers induced an increase in the thermal stability of the materials: the temperature of the maximum degradation rate (T_max_) was 286.6 °C for PHBV/BC and 269.6 °C for PHBV/BC-E, while T_max_ for the unreinforced PHBV compounds was 3–4 °C lower. The changes in the T_on_ values induced by the BC addition were even higher (Table 2).

The opposite effects of TES microspheres and BC nanofibers on the thermal properties of PHBV are also illustrated by the values recorded for the weight loss at 200 °C (WL_200°C_, Table 2). Among the tested samples, the highest and lowest WL_200°C_ were observed for PHBV-E, which lost around 3.26% of its weight, and PHBV/BC, which lost only 1.31%. 

The char residue at 700 °C (R_700°C_) was close to 1 for plasticized PHBV, probably due to the presence of a filler, nucleating agent, or other additives in the PHBV granulate [12]. The addition of the TES blowing agent increased the R_700°C_ value by more than 2 times, while the BC addition had only a small effect on R_700°C_. This was due to the fillers contained in the thermoplastic shell layer of TESs to ensure its strength during expansion, in contrast to the high purity of the BC nanofibers. Indeed, the SEM images displayed in Figure 2 (PHBV-E and PHBV/BC-E) show a lot of white points that correspond to the fillers or other additives present in the composition of the thermoplastic shells of TESs. 

### 3.3. Differential Scanning Calorimetry

**Figure 6:**
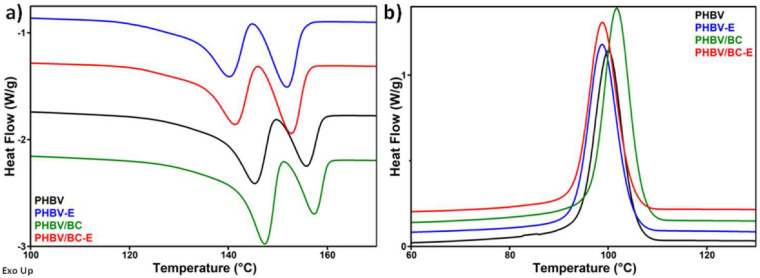
DSC second heating (**a**) and cooling (**b**) curves of PHBV-based materials; curves have been translated for sake of clarity.

The melting and crystallization behaviors of plasticized PHBV after the addition of the TES blowing agent and BC nanofibers were studied using DSC. The DSC curves recorded during the second heating cycle and during cooling are shown in Figure 6, and the characteristic temperatures, together with the corresponding enthalpies, are listed in Table 3. 

Similar to the thermogravimetric results, the blowing agent and BC nanofibers had opposite effects on the thermal behavior of plasticized PHBV. While the blowing agent shifted the melting temperature by 4–6 °C and the crystallization temperature by 1–3 °C toward lower values, the BC nanofibers increased these temperatures by 1.5–2 °C in the absence of TESs, while in the foamed sample, this increase was negligible. The plasticized PHBV used in this work is characterized by a slightly lower melting temperature as compared with PHBV [26], but the differences are significantly higher when the melting temperature of plasticized PHBV is compared to that of PHB or PHB/BC composites [24,38]. Therefore, the addition of TESs enhanced the effect of the plasticizer by further reducing the melting temperature and increasing the processing window [39]. 

Remarkably, the melting-temperature-lowering effect of TESs on PHBV was not observed in the first heating cycle (Table 3). This is due to the fact that the TES microspheres had not expanded during the melt-mixing process of samples, and, therefore, their influence was not yet observed during the first heating cycle. On the contrary, in the first heating cycle, the TES microspheres acted as a filler, increasing the melting temperature of PHBV due to the additives contained in their thermoplastic shells. It is worth mentioning that all of the samples exhibited only one melting peak during the first heating. 

Double melting peaks were noticed for all samples in the second heating cycle. The temperature corresponding to the lower-temperature melting peak is denoted as T_mII,1_, while the higher melting temperature is denoted as T_mII,2_ (Table 3). The occurrence of multiple melting peaks in the DSC thermograms is a common behavior of PHBV [3,24,40]. It is worth remarking that the addition of the blowing agent led to higher enthalpies for the second melting peak (ΔH_II,2_, corresponding to more perfect crystals) in PHBV-E and PHBV/BC-E as compared to the unfoamed samples (Table 3). This indicates that the TESs contributed to the formation of a higher proportion of larger crystals. It is possible that the TES microspheres acted as heterogeneous nucleation agents, favoring the crystallization of PHBV [39]. 

The BC nanofibers and the TES microspheres exhibited different effects on the PHBV crystallinity. While the blowing agent had no significant influence on the degree of crystallinity (X_c_) of the plasticized PHBV, the addition of cellulose nanofibers increased X_c_ from 56.5% for PHBV to 61.1% for the PHBV/BC nanocomposite, acting as a nucleating agent. However, the effect of the BC nanofibers was almost lost in the sample containing both TESs and BC (PHBV/BC-E). A previous study has shown a decrease in the T_m_ and crystallinity of PHBV after the BC addition [41]. This was explained by the harsh treatment of BC during the preparation of BC whiskers. A different effect was reported for plasticized PHB modified with BC nanofibers, where an increase in the crystallization temperature and no significant variation in the T_m_ and X_c_ values were observed [38]. Therefore, the presence of a plasticizer in the PHBV matrix could strongly influence the effect of the reinforcing nanofibers as well as the effect of the blowing agent on the thermal behavior of PHBV. 

### 3.4. Dynamic Mechanical Analysis

**Figure 7:**
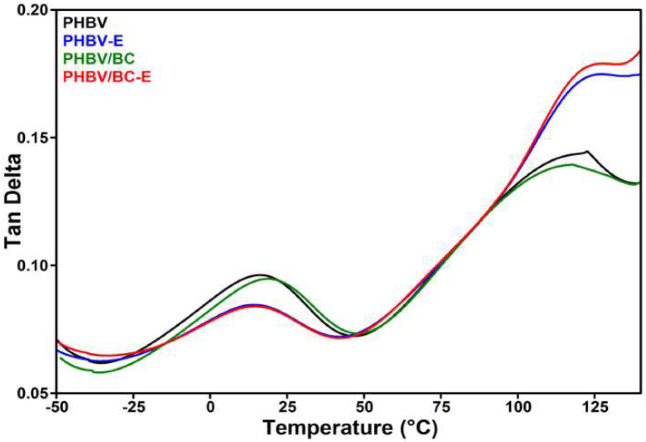
The variation in the mechanical damping factor (tan δ) with temperature.

The changes induced by TESs and BC in the viscoelastic properties of plasticized PHBV were analyzed by DMA over a large temperature range, from −50 °C to 150 °C. The glass-transition temperature (T_g_) corresponding to amorphous PHBV was determined from tan δ vs. temperature curves (Figure 7). The values of the storage modulus (E′) at different temperatures and tan δ (peak value), together with the T_g_ values, are disclosed in Table 4. The storage modulus showed higher values for all samples in the glassy state (before T_g_) and drastically decreased after T_g_ in the rubbery state, similar to the behavior of semi-crystalline polymers. The largest difference in the values of E′ was observed between PHBV and PHBV/BC on the one hand and the corresponding foamed samples on the other hand.

While the addition of BC had only a small influence on the E′ of PHBV, increasing it by 3–8% depending on the temperature, the addition of TESs led to a drop in E’, which was reduced by 4 to 8 times in the same temperature range. A slight increase in the storage modulus was also observed for PHBV/BC nanocomposites containing 3 wt% BC nanowhiskers obtained from the sulfuric acid hydrolysis of BC pellicles [23]. This was a result of the limitations in the polymer chain mobility caused by the nanowhiskers. Conversely, the low E′ values obtained for the foamed samples can be attributed to the increased ductility of the material. The reinforcing capacity of BC was not observed in PHBV/BC-E due to the high mobility of the polymer chains favored by the flexible gas-containing microspheres. In addition, the expanded microspheres prevented the BC nanofibers from forming a network to improve the mechanical properties. Therefore, the cumulative addition of TESs and BC to PHBV/BC-E led to E’ values that did not significantly differ from those of PHBV-E, showing that the addition of the blowing agent had a stronger effect on the mechanical properties of PHBV. However, reducing the size of the expanded TESs and increasing the concentration of BC nanofibers could improve the storage modulus, which is a direction for future work.

The variation in the mechanical damping factor (tan δ) with temperature (Figure 7) shows the occurrence of two peaks for all samples, one at around 15 °C, corresponding to the glass-transition temperature, and the second at around 120 °C, which is generally ascribed to crystal–crystal slippage [24]. A slight increase in T_g_ was obtained after the addition of BC nanofibers to PHBV (PHBV/BC), and a slight decrease in T_g_ was caused by the addition of TESs (PHBV-E and PHBV/BC-E). These T_g_ shifts were determined by the stiffening effect of BC, in opposition to the flexibilizing effect of TESs after foaming. 

### 3.5. Cytotoxicity

**Figure 8:**
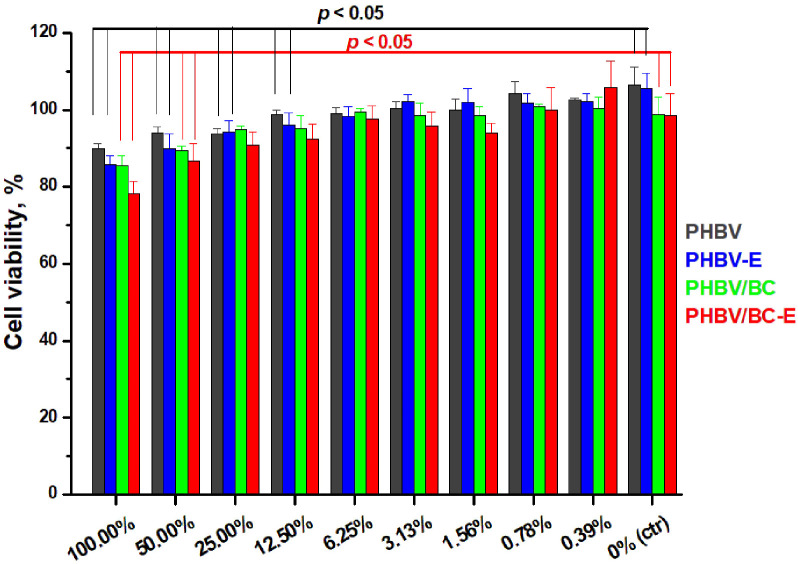
L929 cell viability after 72 h exposure to PHBV, PHBV-E, PHBV/BC, and PHBV/BC-E extracts with different dilutions.

The possible cytotoxic effect of PHBV under the influence of TESs or BC was evaluated using the MTT assay. The cell viability of L929 cells cultivated with the extracts is presented in Figure 8. The cytotoxicity of the PHBV compounds was determined by using an indirect cytotoxicity method. Thus, L929 cells were exposed to the polymer extracts with different dilutions for 72 h.

All PHBV compounds exhibited high cell viability (≥80%), as can be observed in Figure 8. A significant difference (*p* < 0.05) in cell viability as compared to the negative control was observed in the case of the PHBV reference and PHBV-E at dilutions lower than 12.5% (100.0%, 50.0%, 25.0%, and 12.5% extracts). In contrast to these samples, the metabolic activity of L292 cells in indirect contact with nanocomposites containing BC (PHBV/BC and PHBV/BC-E) showed no significant difference when compared to the negative control at a much higher concentration of the extract; thus, cell viability for PHBV/BC and PHBV/BC-E was significantly different (*p* < 0.05) as compared to the negative control only for the 100.0% and 50.0% extracts. 

None of the PHBV compounds showed a significant difference (*p* > 0.05) in cell viability when compared to the PHBV reference, with the exception of PHBV/BC-E. L929 cell viability was significantly lower (*p* < 0.05) when in indirect contact with the PHBV/BC-E nanocomposite as compared to the PHBV reference in the case of the undiluted extracts. 

Given the easy contamination of nanomaterials with endotoxin, originating from the membranes of Gram-negative bacteria [42], a LAL assay was used to determine the concentrations of Gram-negative bacterial endotoxin in the PHBV compounds. The endotoxin concentrations of the samples, determined from a standard curve, were 0.0, 0.0, 0.5, and 0.1 UI/mL for PHBV, PHBV-E, PHBV/BC, and PHBV/BC-E, respectively. It turns out that the cytotoxicity results for the PHBV/BC and PHBV/BC-E specimens could have been influenced by the presence of endotoxin. Indeed, BC could have contained remnants from the culture media and native bacteria scraps, which cause the presence of lipopolysaccharide (LPS), and the lipid part of LPS is responsible for its cytotoxicity [43]. Although BC purification methods must ensure the absence of LPS, the presence of a tiny amount of LPS in BC pellicles could contaminate the BC nanofibers that were used to obtain the PHBV nanocomposites. Special protocols should be considered for the purification of bacterial cellulose so that the cytotoxicity results can be correctly interpreted. 

## 4. Conclusions

In this work, thermally expandable microspheres (TESs) were used as a blowing agent and BC nanofibers were used as a reinforcing agent to correct the properties of PHBV and reduce its cost. PHBV was plasticized with ATBC to reduce the processing temperature and ensure the maximum efficiency of the TES agent. The SEM investigation showed a well-organized porous structure for the foamed PHBV products, with a porosity of around 65% and pores larger than 100 µm in a proportion of 25% for PHBV-E and pores larger than 100 µm in a proportion > 30% for the PHBV/BC-E sample. The presence of open cells and holes, which were observed in the fractured sections of the foamed samples, led to increases in water absorption of 640–650% as compared to unfoamed samples. The TESs and BC had opposite effects on the thermal stability of plasticized PHBV, with TESs decreasing T_max_ by about 17 °C and BC raising it by 3–4 °C. Similarly, while the TES blowing agent shifted the melting temperature by 4–6 °C toward lower values, the BC nanofibers increased these temperatures by 1.5–2 °C. The addition of BC nanofibers also influenced the crystallinity of plasticized PHBV, which increased from 56.5% for neat PHBV to 61.1% for the PHBV/BC nanocomposite. However, this effect was hardly visible in the sample containing both TESs and BC (PHBV/BC-E). The opposite effects of BC and TESs were also observed on the mechanical properties of plasticized PHBV: while the addition of BC increased the E’ of PHBV by 3–8%, depending on the temperature, the addition of TESs reduced E’ by four times at room temperature. Similarly, the stiffening effect of BC led to a slight increase in the T_g_ of PHBV, while the flexibilizing effect of TESs led to a decrease in T_g_. The in vitro cytotoxicity test showed that all PHBV compounds exhibited high cell viability. In contrast to the PHBV reference and the PHBV-E sample, nanocomposites containing BC (PHBV/BC and PHBV/BC-E) showed no significant difference compared to the negative control at a much higher concentration of the extract, showing improved cytotoxicity. The opposite effects of TESs and BC in porous PHBV biocomposites enabled balanced properties, along with lower costs, making PHBV a more attractive biomaterial for engineering, packaging, or medical device applications. 

## Figures and Tables

**Table 1:** Density, porosity (*P*), and average pore size of PHBV-E and PHBV/BC-E.

PHBV Compounds	Density, g cm^−3^	*P* *, %	Average Pore Size, µm
PHBV	1.186 ± 0.004	-	-
PHBV/BC	1.206 ± 0.011	-	-
PHBV-E	0.451 ± 0.012	65.4	75.8 ± 6.7
PHBV/BC-E	0.459 ± 0.010	64.8	81.7 ± 10.9

* *P* = (1 − *ρ_f_*/*ρ_b_)* × 100, where *ρ_f_* is the density of the foam and *ρ_b_* is the bulk density of PHBV (1.305 g cm^−3^ [36]).

**Table 2:** Characteristic parameters for the thermal degradation of PHBV-based materials.

PHBV Compounds	T_on_ °C	T_max_ °C	WL_200°C_ %	R_700°C_ %
**PHBV**	266.8	283.2	1.53	0.958
**PHBV-E**	247.7	265.5	3.26	2.468
**PHBV/BC**	271.3	286.6	1.31	1.240
**PHBV/BC-E**	253.7	269.6	2.38	2.542

**Table 3:** Melting temperatures from the first (T_mI_) and second heating (T_mII,1,2_ corresponding to the double peak) cycles and crystallization temperatures (T_c_), together with the corresponding enthalpies (ΔH_mI_, ΔH_mII,1_, ΔH_mII,2_, and ΔH_c_) and degree of crystallinity (X_c_).

PHBV Compounds	T_mI_ °C	ΔH_mI_ J/g	T_mII,1_ °C	T_mII,2_ °C	ΔH_mII,1_ J/g	ΔH_mII,2_ J/g	T_c_ °C	ΔH_c_ J/g	X_c_ %
PHBV	155.6	58.9	145.3	155.8	40.6	14.8	100.0	50.6	56.5
PHBV-E	158.1	54.5	140.1	151.7	33.2	21.0	98.9	50.5	57.1
PHBV/BC	156.1	61.2	147.3	157.3	44.6	14.0	101.8	53.8	61.1
PHBV/BC-E	157.6	49.4	141.3	152.8	33.7	20.5	98.8	50.5	58.5

**Table 4:** DMA results: storage modulus (E′) values at different temperatures (−30 °C, 30 °C, 75 °C, and 125 °C), glass-transition temperature of PHBV (T_g_), and tan δ at peak T_g_ temperature.

PHBV Compounds	T_g_ °C	tan δ	E_−30°C_ MPa	E_30°C_ MPa	E_75°C_ MPa	E_125°C_ MPa
**PHBV**	16.7	0.10	2428.0	863.4	285.1	57.7
**PHBV-E**	14.1	0.09	549.9	202.4	62.4	8.4
**PHBV/BC**	19.1	0.10	2487.1	904.7	295.1	62.5
**PHBV/BC-E**	14.8	0.09	523.1	195.7	60.0	3.3

## Data Availability

The data presented in this study are available on request from the corresponding author.

## References

[1] Hamad K., Kaseem M., Ayyoob M., Joo J., Deri F. (2018). Polylactic acid blends: The future of green, light and tough. Prog. Polym. Sci..

[2] Da Silva F.A.G.S., Matos M., Dourado F., Reis M.A.M., Branco P.C., Poças F. (2022). Development of a layered bacterial nanocellulose-PHBV composite for food packaging. J. Sci. Food Agric..

[3] Popa M.S., Frone A.N., Panaitescu D.M. (2022). Polyhydroxybutyrate blends: A solution for biodegradable packaging?. Int. J. Biol. Macromol..

[4] Costa S.S., Miranda A.L., de Morais M.G., Costa J.A.V., Druzian J.I. (2019). Microalgae as source of polyhydroxyalkanoates (PHAs)—A review. Int. J. Biol. Macromol..

[5] Policastro G., Panico A., Fabbricino M. (2021). Improving biological production of poly(3-hydroxybutyrate-*co*-3-hydroxyvalerate) (PHBV) co-polymer: A critical review. Rev. Environ. Sci. Biotechnol..

[6] Reddy V.U.N., Ramanaiah S.V., Reddy M.V., Chang Y.-C. (2022). Review of the Developments of Bacterial Medium-Chain-Length Polyhydroxyalkanoates (mcl-PHAs). Bioengineering.

[7] Liu J., Zhao B., Zhang Y., Lin Y., Hu P., Ye C. (2010). PHBV and predifferentiated human adipose-derived stem cells for cartilage tissue engineering. J. Biomed. Mater. Res..

[8] Ibrahim M.I., Alsafadi D., Alamry K.A., Oves M., Alosaimi A.M., Hussein M.A. (2002). A promising antimicrobial bionanocomposite based poly(3-hydroxybutyrate-*co*-3-hydroxyvalerate) reinforced silver doped zinc oxide nanoparticles. Sci. Rep..

[9] Luo J., Zhu M., Wang L., Zhou H., Wen B., Wang X., Zhang Y. (2022). CO_2_-based fabrication of biobased and biodegradable poly (3-hydroxybutyrate-*co*-3-hydroxyvalerate)/graphene nanoplates nanocomposite foams: Toward EMI shielding application. Polymer.

[10] El-hadi A.M., AbdElbary A.M., Alluqmani S.M. (2019). The role of polyaniline and plasticizer on the development of the electrical conductivity of PHB composites. J. Compos. Mater..

[11] Nosal H., Moser K., Warzała M., Holzer A., Stanczyk A., Sabura E. (2021). Selected Fatty Acids Esters as Potential PHB-V Bioplasticizers: Effect on Mechanical Properties of the Polymer. J. Polym. Environ..

[12] Panaitescu D.M., Nicolae C.A., Frone A.N., Chiulan I., Stanescu P.O., Draghici C., Iorga M., Mihailescu M. (2017). Plasticized poly(3-hydroxybutyrate) with improved melt processing and balanced properties. J. Appl. Polym. Sci..

[13] Chaos A., Sangroniz A., González A., Iriarte M., Sarasua J.R., del Río J., Etxeberria A. (2018). Tributyl citrate as an effective plasticizer for biodegradable polymers: Effect of the plasticizer on the free volume, transport and mechanical properties: Tributyl citrate as an effective plasticizer for biodegradable polymers: Effect of the plasticizer on the free volume, transport and mechanical properties. Polym. Int..

[14] Choi J.S., Park W.H. (2004). Effect of biodegradable plasticizers on thermal and mechanical properties of poly(3-hydroxybutyrate). Polym. Test..

[15] Usurelu C.D., Badila S., Frone A.N., Panaitescu D.M. (2022). Poly(3-hydroxybutyrate) Nanocomposites with Cellulose Nanocrystals. Polymers.

[16] Do Amaral Montanheiro T.L., Montagna L.S., Patrulea V., Jordan O., Borchard G., Monteiro Lobato G.M., Catalani L.H., Lemes A.P. (2019). Evaluation of cellulose nanocrystal addition on morphology, compression modulus and cytotoxicity of poly(3-hydroxybutyrate-*co*-3-hydroxyvalerate) scaffolds. J. Mater. Sci..

[17] Haldar D., Purkait M.K. (2020). Micro and nanocrystalline cellulose derivatives of lignocellulosic biomass: A review on synthesis, applications and advancements. Carbohydr. Polym..

[18] Singhania R.R., Patel A.K., Tseng Y.S., Kumar V., Chen C.W., Haldar D., Saini J.K., Dong C.D. (2022). Developments in bioprocess for bacterial cellulose production. Bioresour. Technol..

[19] Panaitescu D.M., Frone A.N., Chiulan I., Casarica A., Nicolae C.A., Ghiurea M., Trusca R., Damian C.M. (2016). Structural and morphological characterization of bacterial cellulose nano-reinforcements prepared by mechanical route. Mater. Des..

[20] Lahiri D., Nag M., Dutta B., Dey A., Sarkar T., Pati S., Edinur H.A., Zulhisyam A.K., Noor Haslina M.N., Ray R.R. (2021). Bacterial Cellulose: Production, Characterization, and Application as Antimicrobial Agent. Int. J. Mol. Sci..

[21] Mishra S., Singh P.K., Pattnaik R., Kumar S., Ojha S.K., Srichandan H., Parhi P.K., Jyothi R.K., Sarangi P.K. (2022). Biochemistry, Synthesis, and Applications of Bacterial Cellulose: A Review. Front. Bioeng. Biotechnol..

[22] Oprea M., Panaitescu D.M., Nicolae C.A., Gabor A.R., Frone A.N., Raditoiu V., Trusca R., Casarica A. (2020). Nanocomposites from functionalized bacterial cellulose and poly(3-hydroxybutyrate-*co*-3-hydroxyvalerate). Polym. Degrad. Stab..

[23] Ambrosio-Martin J., Fabra M.J., López-Rubio A., Gorassi G., Sorrentino A., Lagaron J.M. (2016). Assessment of Ball Milling as a Compounding Technique to Develop Nanocomposites of Poly(3-Hydroxybutyrate-*co*-3-Hydroxyvalerate) and Bacterial Cellulose Nanowhiskers. J. Polym. Environ..

[24] Panaitescu D.M., Frone A.N., Chiulan I., Nicolae C.A., Lupescu I. (2018). Role of bacterial cellulose and poly (3-hydroxyhexanoate-*co*-3-hydroxyoctanoate) in poly (3-hydroxybutyrate) blends and composites. Cellulose.

[25] Codreanu A., Balta C., Herman H., Cotoraci C., Mihali C.V., Zurbau N., Zaharia C., Rapa M., Stanescu P., Radu I.C. (2020). Bacterial Cellulose-Modified Polyhydroxyalkanoates Scaffolds Promotes Bone Formation in Critical Size Calvarial Defects in Mice. Materials.

[26] Panaitescu D.M., Trusca R., Gabor A.R., Nicolae C.A., Casarica A. (2020). Biocomposite foams based on polyhydroxyalkanoate and nanocellulose: Morphological and thermo-mechanical characterization. Int. J. Biol. Macromol..

[27] Mendonça R.H., Meiga T.D., Costa M.F., Thiré R.M. (2013). Production of 3D scaffolds applied to tissue engineering using chitosan swelling as a porogenic agent. J. Appl. Polym. Sci..

[28] Oluwabunmi K.E., Zhao W., D’Souza N.A. (2021). Carbon Capture Utilization for Biopolymer Foam Manufacture: Thermal, Mechanical and Acoustic Performance of PCL/PHBV CO_2_ Foams. Polymers.

[29] Xu J.K., Zhang L., Li D.L., Bao J.B., Wang Z.B. (2020). Foaming of Poly(3-hydroxybutyrate-*co*-3-hydroxyvalerate) with Supercritical Carbon Dioxide: Foaming Performance and Crystallization Behavior. ACS Omega.

[30] Kmetty Á., Litauszki K. (2020). Development of Poly (Lactide Acid) Foams with Thermally Expandable Microspheres. Polymers.

[31] Gelin K., Bodin A., Gatenholm P., Mihranyan A., Edwards K., Strømme M. (2007). Characterization of water in bacterial cellulose using dielectric spectroscopy and electron microscopy. Polymer.

[32] Gong P., Taniguchi T., Ohshima M. (2014). Nanoporous structure of the cell walls of polycarbonate foams. J. Mater. Sci..

[33] Scandola M., Focarete M.L., Adamus G., Sikorska W., Baranowska I., Świerczek S., Gnatowski M., Kowalczuk M., Jedliński Z. (1997). Polymer blends of natural poly(3-hydroxybutyrate-*co*-3-hydroxyvalerate) and a synthetic atactic poly(3-hydroxybutyrate). Characterization and biodegradation studies. Macromolecules.

[34] Do Amaral Montanheiro T.L., Montagna L.S., Patrulea V., Jordan O., Borchard G., Ribas R.G., Campos T.M.B., Thim G.P., Lemes A.P. (2019). Enhanced water uptake of PHBV scaffolds with functionalized cellulose nanocrystals. Polym. Test..

[35] Wright Z., Frank C. (2014). Increasing Cell Homogeneity of Semicrystalline, Biodegradable Polymer Foams with a Narrow Processing Window via Rapid Quenching. Polym. Eng. Sci..

[36] Kunyu Z., Manjusri M., Mohanty A. (2014). Toughened Sustainable Green Composites from Poly(3-hydroxybutyrate-*co*-3-hydroxyvalerate) Based Ternary Blends and Miscanthus Biofiber. ACS Sustain. Chem. Eng..

[37] Shi Z., Zhao G., Zhang L., Wang G., Chai J. (2022). Ultralight and hydrophobic PVDF/PMMA open-cell foams with outstanding heat-insulation and oil-adsorption performances fabricated by CO_2_ molten foaming. J. CO_2_ Util..

[38] Panaitescu D.M., Ionita E.R., Nicolae C.-A., Gabor A.R., Ionita M.D., Trusca R., Lixandru B.-E., Codita I., Dinescu G. (2018). Poly(3-hydroxybutyrate) Modified by Nanocellulose and Plasma Treatment for Packaging Applications. Polymers.

[39] Umemura R.T., Felisberti M.I. (2021). Plasticization of poly(3-hydroxybutyrate) with triethyl citrate: Thermal and mechanical properties, morphology, and kinetics of crystallization. J. Appl. Polym. Sci..

[40] Shan G.F., Gong X., Chen W.P., Chen L., Zhu M.F. (2011). Effect of multi-walled carbon nanotubes on crystallization behavior of poly(3-hydroxybutyrate-*co*-3-hydroxyvalerate). Colloid. Polym. Sci..

[41] Ting Z.X., Yan L.J. (2022). Effects of Bacterial Cellulose Whisker Melting Composite on Crystallization and Mechanical Properties of PHBV Composites. Macromol. Res..

[42] Li Y., Fujita M., Boraschi D. (2017). Endotoxin Contamination in Nanomaterials Leads to the Misinterpretation of Immunosafety Results. Front. Immunol..

[43] Dourado F., Gama M., Rodrigues A.C. (2017). A Review on the toxicology and dietetic role of lupe cellulose. Toxicol. Rep..

